# Impact of an Enhanced Disinfection Protocol on the Incidence of *Clostridioides difficile* Infections and Antibiotic Consumption in a Hospital Setting: A Retrospective Intervention Study

**DOI:** 10.3390/jcm14144904

**Published:** 2025-07-10

**Authors:** Patryk Tarka, Wiesław Hreczuch, Arkadiusz Chruściel, Michał Piotrowski, Anna Olczak-Pieńkowska, Karol Warda, Daniel Rabczenko, Krzysztof Kanecki, Aneta Nitsch-Osuch

**Affiliations:** 1 Department of Social Medicine and Public Health, Medical University of Warsaw, 02-106 Warsaw, Poland; krzysztof.kanecki@wum.edu.pl (K.K.); aneta.nitsch-osuch@wum.edu.pl (A.N.-O.); 2 MEXEO, 47-225 Kedzierzyn-Kozle, Poland; mexeo@mexeo.pl (W.H.); arkach@mexeo.pl (A.C.); 3 Department of Medical Microbiology, Medical University of Warsaw, 02-091 Warsaw, Poland; piotrowski.michal90@gmail.com; 4 Department of Public Health, Epidemiology and Vaccinology, Military Institute of Medicine—National Research Institute, 04-141 Warsaw, Poland; aolczak-pienkowska@wim.mil.pl (A.O.-P.); kwarda@wim.mil.pl (K.W.); 5 Department of Population Heath Monitoring and Analyses, National Institute of Public Health—National Institute of Hygiene, 00-791 Warsaw, Poland; drabczenko@pzh.gov.pl

**Keywords:** *Clostridioides difficile*, sporicidal activity, chlorine dioxide, EN 17126, EN 17846

## Abstract

**Background**: *Clostridioides difficile* infection (CDI) is a major concern in hospital-acquired infections. *C. difficile* spores can survive on surfaces for months and require sporicidal disinfection for elimination. The use of disinfectants should be based on laboratory-confirmed sporicidal activity, tested according to current standards in suspension and carrier tests. Further evaluation of disinfectant efficacy should occur in clinical settings by analyzing reductions in CDI incidence. This study aims to conduct a retrospective analysis of the impact of a new disinfection protocol and concurrent changes in antibiotic consumption on the incidence of healthcare-acquired CDI (HA-CDI). **Methods**: This retrospective, single-center study assessed the impact of a chlorine dioxide-based disinfection protocol on HA-CDI across three periods: pre-intervention, intervention, and post-intervention. An interrupted time series analysis (ITS) with a Poisson distribution was used to evaluate the incidence of HA-CDI, while antibiotic consumption data were analyzed to identify any correlation with CDI infection rates. **Results**: Incidence Rate Ratio (IRR) before the intervention is 1.00, serving as the reference value. During the intervention period, the IRR is 0.79 (95% CI: 0.42–1.36; *p* = 0.43), indicating a decrease in the incidence of infections compared to the pre-intervention period, although this result is not statistically significant. After the intervention, the IRR is 0.53 (95% CI: 0.26–0.97; *p* = 0.057), suggesting a further reduction in the incidence of CDI; this result is on the borderline of statistical significance (*p* = 0.057), indicating a potential effect of the intervention, albeit without full statistical certainty. **Conclusions**: The absence of a CDI surge despite increased antibiotic consumption highlights the synergistic relationship between antibiotic stewardship and rigorous infection control practices. The combination of the improved disinfection protocol and comprehensive staff training proved remarkably effective in mitigating CDI risk. Cleaning and disinfection in healthcare facilities is crucial for the prevention of healthcare-associated infections.

## 1. Background

*Clostridioides difficile* infections (CDI) pose a significant challenge in hospital settings, leading to increased morbidity, prolonged hospital stays, and rising healthcare costs [1,2,3]. These infections are predominantly associated with the use of antibiotics, which disrupt normal gut flora and facilitate the overgrowth of *C. difficile*. The epidemiology of CDI has shown alarming trends, particularly in hospitalized patients, who are often exposed to multiple antibiotic treatments for various underlying conditions [4,5]. Consequently, implementing effective strategies for prevention and control is highly important.

One of the most critical components in controlling CDI outbreaks is maintaining rigorous hygiene practices and effective disinfection protocols within healthcare facilities. Inadequate cleaning and disinfection of surfaces can lead to the persistence of *C. difficile* spores in the hospital environment, contributing to ongoing transmission among patients [6,7]. Traditional cleaning methods have been called into question, imposing the exploration of new disinfectants that have shown efficacy against *C. difficile* spores. The introduction of chlorine dioxide-based products represents a promising approach to enhancing environmental hygiene. The efficacy of surface disinfection agents, including sporicidal agents, should be based on a hierarchy of evidence confirming their effectiveness. Technical Committee 216 (TC 216) “Chemical Disinfectants and Antiseptics” of the European Committee for Standardization (CEN) has been developing test methods for evaluating the efficacy of disinfectants in Europe since 1989 [8]. TC 216 introduced a three-phase model for testing chemical disinfectants and antiseptics [9], which is summarized below:

- Phase 1 tests (suspension tests) are conducted to determine whether a chemical disinfectant or antiseptic possesses bactericidal, fungicidal, yeasticidal, or sporicidal activity, regardless of its specific area of application. Phase 1 tests cannot be used to support any product-related claims.
- Phase 2/step 1 tests use quantitative suspension methods in which microorganisms are exposed to chemical disinfectants or antiseptics at various concentrations, contact times, and temperatures, often in the presence of interfering substances. These tests confirm product activity under laboratory conditions that resemble the intended use (e.g., on instruments or surfaces, with or without mechanical action, in medical settings). An example of a Phase 2/step 1 standard is EN 17126, which assesses sporicidal activity [10].
- Phase 2/step 2 tests are based on carrier methods conducted under conditions that simulate practical use. For sporicidal activity in the medical area, a carrier-based standard involving mechanical action—EN 17846 [11]—is available. This document applies to four methods of product application for wiping and/or mopping:
  (a) soaking any non-specified wipe or mop with the product;
  (b) spraying the product onto any non-specified or specified wipe and/or mop;
  (c) user impregnation of specified wipes or mops with the product according to the manufacturer’s instructions;
  (d) pre-impregnation of specified wipes or mops by the manufacturer as ready-to-use products.

Practical efficacy tests of disinfectants should complement suspension and carrier tests in order to enhance the safety of daily clinical practice for both healthcare professionals and patients. Sporicidal surface disinfection is an effective component of hospital transmission control bundles and is recommended both in outbreak scenarios and under endemic conditions [12]. McDonald LC et al. [13] proposed an evidence-based hierarchy for evaluating disinfection strategies, starting with the foundation (i.e., Level I) of laboratory efficacy studies. Further assessment of the disinfectant’s effectiveness should be conducted in clinical settings through analysis of incidence reduction. Many sporicidal disinfectants have characteristics that discourage use [14]. Sodium hypochlorite causes irritation, nausea, vomiting, dizziness, breathing distress, headache, and cough. At high concentrations, hypochlorite may cause upper respiratory tract edema and pulmonary edema [15]; peracetic acid can cause nasal problems, watery eyes, asthma-like symptoms, and skin problems [16]; and glutaraldehyde can cause respiratory tract irritation, asthma, and chronic obstructive pulmonary disease [17].

In this context, chlorine dioxide is of great interest. Chlorine dioxide is an inert chlorine compound. It differs from elemental chlorine both in terms of chemical structure and behavior [18]. Because of rapid decomposition, its oxidizing effect on the skin is milder than that of sodium hypochlorite, even at very high concentrations [19].

Furthermore, the rational use of antibiotics plays a crucial role in CDI prevention [20]. Identifying and restricting the use of high-risk antibiotics—such as carbapenems, clindamycin, third-generation cephalosporins, and fluoroquinolones—is essential in mitigating CDI risks. Monitoring antibiotic consumption and assessing its correlation with CDI rates can provide valuable insights for optimizing antibiotic stewardship programs.

This study aims to conduct a retrospective analysis of the impact of a new disinfection protocol and concurrent changes in antibiotic consumption on the incidence of healthcare-acquired CDI (HA-CDI). By analyzing data collected over a study period of before, during, and after the implementation of the intervention, this research aspires to shed light on effective strategies for combating CDI in a hospital environment, ultimately contributing to improved patient care and safety.

The primary objective of this study was to evaluate the effectiveness of a new disinfection protocol using chlorine dioxide-based products on the incidence of HA-CDI in a clinical setting, with rate measured via the incidence rate ratio (IRR) across different time periods.

## 2. Methodology

### 2.1. Study Aims

To describe the incidence rate ratio for hospital-acquired infections (HAIs), we used incidence of hospital-acquired infections per 1000 patient-days, which is a standard measure for HAI. We calculated it as the number of new HAIs divided by the total number of patient-days (often expressed per 1000 patient-days) in the analyzed period. This measure allows for standardized comparison across different settings and time periods, accounting for variations in patient numbers and lengths of stay [21].

This is a measure recommended by The European Centre for Disease Prevention and Control (ECDC): it recommends using the incidence density of healthcare-associated infections (HAIs) expressed as the number of HAI episodes per 1000 patient-days for surveillance in hospitals [22].

Secondary objectives included the following:

- To determine the impact of the disinfection intervention on infection rates and antibiotic stewardship within the clinical settings observed;
- To compare antibiotic consumption trends (in defined daily doses per 1000 patient-days) before, during, and after the implementation of the disinfection intervention;
- To identify any correlations between specific antibiotics linked to an increased risk of CDI and the infection rates, in order to inform future antibiotic stewardship programs.

### 2.2. Study Design and Setting

This was a retrospective, single-center study conducted in the Department of Gastroenterology, the Department of Anesthesiology and Intensive Care, and the Department of Internal Medicine, Nephrology, and Dialysis Therapy with the Dialysis Unit Military Institute of Medicine—National Research Institute. The wards were selected based on the highest numbers of HA-CDI cases.

Outcome measures were reviewed for the period from January 2019 to October 2023, which was divided into the following intervals: pre-intervention period (January 2019 to July 2022), intervention period (August 2022 to January 2023), and post-intervention period (March 2023 to October 2023). The primary endpoint was the HA-CDI rate per 10,000 patient-days during the post-intervention period.

To evaluate clinical effectiveness, an interrupted time series (ITS) analysis with a Poisson distribution was used to compare the incidence of HA-CDI across the pre-intervention, intervention, and post-intervention periods. High-touch environmental surfaces (HITES) were assessed by epidemiological nurses and with the use of adenosine triphosphate bioluminescence, which measures the amount of light generated by chemical reaction, and produces a result expressed in Relative Light Units (RLUs). The intensity of the light is proportional to the amount of ATP and therefore the degree of contamination. Environmental surface Adenosine triphosphate (ATP) bioluminescence levels were monitored using the NGI Luminometer Clean-Trace ATP Surface Test, manufactured by 3M™ Health Care in St. Paul, Minnesota, USA, and swabs (3 M™ Clean-Trace™ Surface ATP Test Swab). Collection was carried out according to the instructions provided by the manufacturer [23]. All samples were collected 30 min after disinfection to allow the sporicidal activity to be completed and the surfaces to dry, thereby minimizing potential interference between residual chemical substances and the bioluminescence reaction [24].

The manufacturer defines a negative (clean) sample as a RLU level ≤ 250 RLUs/100 cm^2^ [23]; in this study, all surfaces with ≥100 RLUs/100 cm^2^ after disinfection were considered contaminated and thus re-cleaned, based on a review of ATP-bioluminescence in healthcare environments which proposed a cutoff of 100 RLU/100 cm^2^ in high-risk environments [25,26]. The surfaces examined in the study are listed in the Supplementary Materials.

### 2.3. Disinfection Preparations

Three chlorine-dioxide-based surface disinfectants were tested:

Product 1, ARMEX 5 MD, is a two-component cleaning disinfectant concentrate for large surfaces, which, after dilution, is applied at a concentration of ClO_2_ of 100 ppm, in combination with an activator consisting of acetic acid 25% with 20% surfactants.

Product 2, ARMEX 5 Foam, is a ready-to-use foam used in combination with 1500 ppm chlorine dioxide (0.15%) with citric acid 2.5% (activator) and 1% surfactant.

Product 3, ARMEX 5 WC, is a ready-to-use gel in combination with 1500 ppm chlorine dioxide with citric acid 2.5% (activator) and 1% surfactant.

For Product 1, ARMEX 5 MD, the aqueous solution of activated chlorine dioxide for surface disinfection was obtained by mixing an adequate volume with tap water. A CIO_2_ concentration of 100 ppm in the solution was obtained by adding 25 mL of the precursor and 25 mL of the activator to 5 L of tap water. All products met the European Union standards, including suspension tests (EN 17126) [10] and carrier tests (EN 17846) [11], registered by the Office for Registration of Medicinal Products, Medical Devices and Biocidal Products [27].

### 2.4. Enhanced Disinfection Protocol

The intervention consisted of education on cleaning techniques, and auditing of cleaning thoroughness with feedback to staff. A multimodal approach to environmental cleaning in healthcare facilities was used, encompassing five key strategies: the product (effective in both suspension and carrier tests); the cleaning approach, technique, education (visual posters showing high-touch surfaces), and training; audit (ATP monitoring); feedback; and communication [28]. Following the implementation of an improved disinfection protocol, patient rooms and their equipment were cleaned and disinfected daily using chlorine dioxide-based products.

The product Armex 5 MD, used in concentrate form (precursor) along with an activator, was diluted at a ratio of 25 mL to 5 L of cold tap water to achieve a final concentration of 100 ppm. Floor surfaces were then cleaned using flat mops with a figure-eight motion, and the disinfectant was left on the surface for a 15 min contact time. For sink drains, 250 mL of the 100 ppm solution was poured in and left for 15 min. In the case of toilet bowl traps, 25 mL of Armex 5 MD concentrate was poured directly into the siphon.

Armex 5 Foam, in foam form, was applied in two doses directly onto the surface to be cleaned and disinfected, then spread using a disposable dry wipe. The product was left for a contact time of 5 min.

Armex 5 WC, in gel form, was applied directly onto the surface to be cleaned and disinfected to ensure complete coverage of the inner walls of the toilet bowl and was also left for a 5 min contact time.

Cleaning and disinfection were performed on high-touch environmental surfaces (HITES) in accordance with Centers for Disease Control and Prevention (CDC) guidelines [29]. These included the chair, room sink, room light switch, inner doorknob, bed rails/controls, tray table, IV pole (grab area), call box/button, telephone, and toilet seat. Additionally, floors, sink drains, and toilet bowl traps were also cleaned and disinfected.

#### Data Collection Method and Tools

Antibiotic consumption was expressed in defined daily doses per 1000 patient-days (PD). The PD measure refers to the patients’ stay on a ward or day ward and is approved by National Health Fund for contracting health services. Analysis of antimicrobial (AB) consumption included ABs that may pose a risk of causing CDI infections. Those were amoxicillin (J01CA04), carbapenems (J01DH), third-generation cephalosporins (J01DD), fluoroquinolones (J01MA), and clindamycin (J01FF01). Both PDs and data on antibiotics distributed to the selected wards were obtained from the hospital’s electronic system in monthly intervals. The data on the numbers and contents of packages were used in calculations to express AC in defined daily doses (DDDs) according to the methodology developed by the World Health Organization Collaborating Centre for Drug Statistics Methodology and recommended for drug utilization studies. The trends of consumption for the ABs mentioned were observed during the period of study to look for possible relations with CDI infections.

### 2.5. Statistical Analysis

Interrupted time series analysis [30] was performed to assess changes in the incidence of HO-CDI and the use of individual broad-spectrum antimicrobials before and after the interventions. Furthermore, Poisson regression analysis was used to determine whether the antimicrobials were independent factors affecting HO-CDI [31]. Predictive values are presented as incidence rate ratios (IRRs) with 95% confidence intervals (CI). In all analyses, two-tailed *p*-values < 0.05 were considered statistically significant, and all statistical analyses were conducted using R Statistical Software (version 4.3.1; R Foundation for Statistical Computing, Vienna, Austria) [32].

## 3. Results

### 3.1. CDI Prevalence During Study Period

Poisson regression results for the incidence of *C. difficile* infections before and after the intervention are expressed as an Incidence Rate Ratio (IRR). The IRR before the intervention is 1.00, serving as the reference value. During the intervention period, the IRR is 0.79 (95% CI: 0.42–1.36; *p* = 0.43), indicating a decrease in the incidence of infections compared to the pre-intervention period, although this result is not statistically significant. After the intervention, the IRR is 0.53 (95% CI: 0.26–0.97; *p* = 0.057), suggesting a further reduction in the incidence *of C. difficile* infections; this result is on the borderline of statistical significance (*p* = 0.057), indicating a potential effect of the intervention, albeit without full statistical certainty.

The monthly distribution of *C. difficile* infection cases across the three observation periods was assessed separately for the anesthesiology, gastroenterology, and internal medicine departments (Table 1, Table 2 and Table 3). The analysis did not reveal statistically significant differences between the periods in any of the departments (Fisher’s exact test: anesthesiology, *p* = 0.79; gastroenterology, *p* = 0.10; internal medicine, *p* = 0.77). In the gastroenterology department, a reduction in infection frequency was observed in the most recent period, with no infections recorded across all months, although this trend did not reach statistical significance.

### 3.2. Gastroenterology

**Figure 1:**
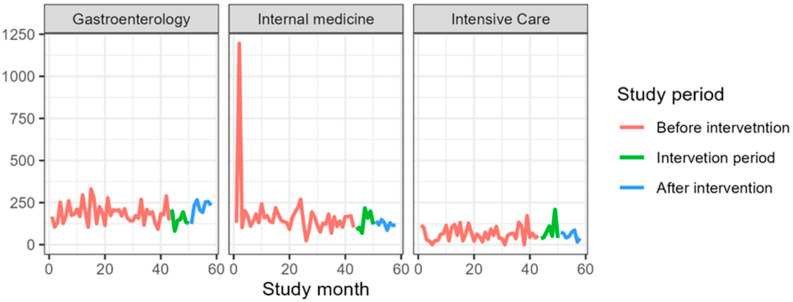
Fluoroquinolones consumption during the study period expressed in DDS/1000PD.

**Figure 2:**
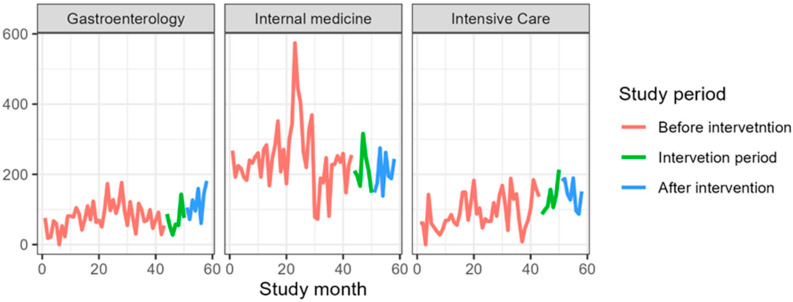
Cephalosporins consumption during the study period expressed in DDS/1000PD.

**Figure 3:**
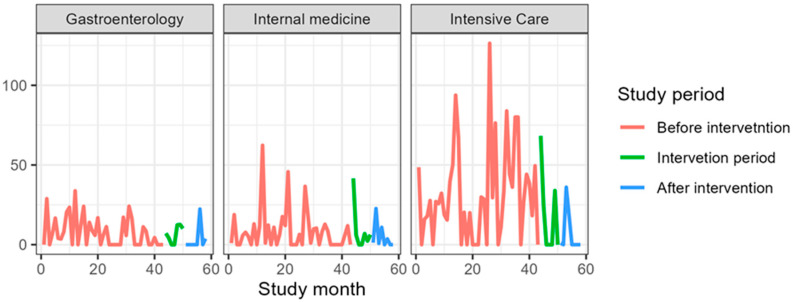
Amoxicillin consumption during the study period expressed in DDS/1000PD.

**Figure 4:**
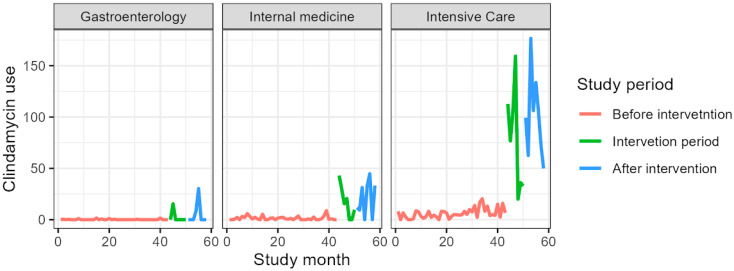
Clindamycin consumption during the study period expressed in DDS/1000PD.

**Figure 5:**
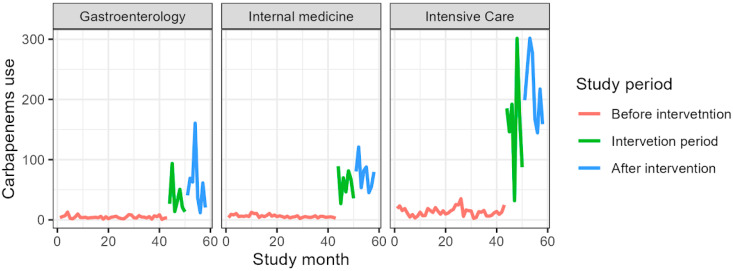
Carbapenems consumption during the study period expressed in DDS/1000PD.

Table 4 presents antibiotic consumption in the gastroenterology clinic as DDDs per 1000 PD. The consumption of all included antibiotics increased, except for amoxicillin, which decreased from a mean of 7.7 DDDs/1000 PD before the intervention to 3.7 DDDs/1000 PD. The largest increase was observed for carbapenems, rising from a mean of 4.6 DDDs/1000 PD before the intervention to 57.9 DDDs/1000 PD (Figure 1, Figure 2, Figure 3, Figure 4 and Figure 5).

### 3.3. Internal Medicine Department

Table 5 presents antibiotic consumption in the gastroenterology clinic as DDDs per 1000 PD. Increased consumption of clindamycin and carbapenems was observed after the intervention, with the largest increase seen for carbapenems, rising from a mean of 6.0 DDDs/1000 PD before the intervention to 75.6 DDDs/1000 PD. Decreased consumption was noted for fluoroquinolones, cephalosporins, and amoxicillin, with the largest reduction observed for fluoroquinolones, which decreased from a mean of 171.4 DDDs/1000 PD before the intervention to 123.2 DDDs/1000 PD after the intervention (Figure 1, Figure 2, Figure 3, Figure 4 and Figure 5).

### 3.4. Department of Anesthesiology and Intensive Care

Table 6 presents antibiotic consumption in the Department of Anesthesiology and Intensive Care as DDDs per 1000 PD. The consumption of all included antibiotics increased, except for fluoroquinolones and amoxicillin. The largest increase was observed for carbapenems, rising from a mean of 13.4 DDDs/1000 PD before the intervention to 214.6 DDDs/1000 PD (Figure 1, Figure 2, Figure 3, Figure 4 and Figure 5).

### 3.5. Overall Antibiotic Consumption

Table 7 presents regression results for antibiotic consumption before and after the intervention. For clindamycin, a significant increase in consumption was observed, with the beta coefficient rising to 39.5 (95% CI: 30.2, 48.7; *p* < 0.001) after the intervention. Carbapenems also showed a substantial increase, with beta coefficients increasing to 108.0 (95% CI: 92.4, 123.7; *p* < 0.001) after the intervention. In contrast, amoxicillin consumption decreased significantly after the intervention, with beta decreasing to −10.9 (95% CI: −19.1, −2.7; *p* = 0.009) after the intervention. There were no significant changes observed for fluoroquinolones (beta: −7.2; 95% CI: −48.4, 34.0; *p* = 0.7) or cephalosporins (beta: 16.9; 95% CI: −10.2, 44.0; *p* = 0.2).

## 4. Discussion

This retrospective study evaluated the impact of replacing a sodium dichloroisocyanurate (NaDCC)-based disinfectant with chlorine dioxide-based formulations in three variations on the incidence of CDI. The sporicidal activity of these disinfectants was assessed according to European Union standards, including suspension tests (EN 17126) [10] and carrier tests (EN 17846) [11].

The implementation of a comprehensive infection prevention and control strategy is crucial in mitigating the spread of healthcare-associated infections, including CDI. While antibiotic use remains a significant risk factor for CDI, it is not the sole determinant of infection rates. This study demonstrates that a multifaceted approach, incorporating enhanced environmental disinfection protocols alongside comprehensive staff training, can effectively control CDI incidence even in the context of increased antibiotic consumption. The importance of staff training in preventing CDI is also supported by other reports and recommendations [33,34].

Recommendations for the practical application of disinfectants can be drawn from suspension test results only to a limited extent, as the homogeneous conditions in suspension tests rarely reflect real-world scenarios [35]. The results of published studies on sporicidal activity using the four-field carrier test [36] demonstrate that manufacturer claims regarding sporicidal agents, often based solely on suspension tests, do not necessarily guarantee that surface disinfectants exhibit sufficient sporicidal efficacy against *C. difficile* spores under practical conditions. To address this, an appropriate testing method was developed to evaluate the efficacy of chemical disinfectants against *C. difficile* spores under simulated practical conditions, including a reliable protocol for preparing *C. difficile* spores [37]. This formed the basis for the EN 17846 standard [11]. The PVC carrier material used in the four-field method is the most challenging surface to disinfect under medical conditions, representing a worst-case scenario for simulating disinfected surfaces in healthcare settings [38]. While smooth surfaces made of stainless steel and plastics theoretically facilitate effective disinfection, the presence of viable spores on both steel and plastic surfaces treated with sporicidal disinfectants contradicts this hypothesis, clearly demonstrating the resistance of spores to disinfectants [39]. Factors influencing this resistance may include both the hydrophobicity of the surfaces and the spores themselves.

In studies by Joshi LT, the hydrophobicity of spore surfaces varied significantly and was correlated with their ability to adhere to stainless steel surfaces [40]. The surface tension of disinfectant solutions is often too high to effectively and fully cover and wet the disinfected surfaces. Nonionic compounds are the most effective at reducing water surface tension to the range of 25–35 millinewton/metre (mN/m) [41], which is why they are included in the tested chlorine dioxide formulations. Sodium dichloroisocyanurate (NaDCC) solutions, on the other hand, have a high surface tension and are incompatible with nonionic surfactants. The compositions are not stable with respect to the active chlorine content; that is, within a very short time the starting available chlorine content decreases to such an extent that for all practical purposes the detergent compositions are chlorine-free [42]. The low wettability of NaDCC at a concentration of 10,000 ppm active chlorine results in its inability to achieve the required reduction against *C. difficile* spores in the four-field test. However, a disinfectant with the same active substance (NaDCC) and concentration (10,000 ppm active chlorine), but supplemented with an anionic surfactant, demonstrated sporicidal activity against *C. difficile* in the four-field method [43]. The spectrum of activity of active microbiocidal substances or classes of compounds (e.g., chlorine compounds, peracetic acid, aldehydes) used for sporicidal disinfection serves only as a general guideline [44]. Product formulations can influence the effectiveness of the disinfectant [45]. Wiping alone may contribute to the spread of *C. difficile* spores on disinfected surfaces [46]. Lawley [47] demonstrated in a mouse experiment that as few as five to ten *C. difficile* spores per cm^2^ (125–250 CFU/25 cm^2^) are sufficient to infect 50% of mice within one hour of the median infective dose (ID50). Therefore, the maximum spread of 50 CFU on fields 2–4, as specified in the EN 17846 standard, appears appropriate. The tested disinfectants did not transfer spores to other surfaces. Since siphons in medical facilities are an important risk factor for the development of infections caused by *C. difficile* [48], sporicidal disinfection of toilet, sink, and shower siphons was also performed using chlorine dioxide-based products. The four-field test method describes the disinfection process across four designated areas, beginning with the contaminated field 1, followed by fields 2–4, and returning to the starting point. It is currently included in standard EN 16615 [49], which outlines the inactivation and transfer of vegetative bacteria and yeasts during disinfection involving mechanical action. The four-field test method is also incorporated into EN 17846, which evaluates sporicidal activity against *C. difficile*. In the future, this method is expected to be expanded to include mycobacteria, fungi, and viruses [50]. These methods are robust and reproducible, providing professional users with the assurance that the products have been tested according to standardized procedures.

Moreover, a noted increase in antibiotic utilization within the studied period, that did not translate into a corresponding surge in CDI cases, might also be an effect of the fact that the introduction of a novel disinfection protocol, utilizing chlorine dioxide-based solutions, enhanced the effectiveness of environmental cleaning. This targeted approach, focusing on the elimination of *C. difficile* spores from high-touch surfaces and patient environments, might directly reduce the environmental reservoir of infection.

Furthermore, a comprehensive training program for infection control personnel was implemented concurrently with the new protocol. This initiative emphasized the importance of meticulous adherence to the new disinfection procedures, proper hand hygiene techniques, and appropriate use of personal protective equipment (PPE). The training program demonstrably raised awareness among staff regarding the transmission dynamics of CDI and the vital role of careful adherence to infection control protocols in preventing the spread of infection. This increased awareness and consistent application of updated procedures might have contributed to a lower rate of transmission, despite the higher rate of antibiotic use.

Another factor contributing to the success of the new protocol might also be the superior organoleptic profile of the chlorine dioxide-based disinfectant. Staff reported an improvement in the product’s ease of use and a more pleasant scent compared to the previously used disinfectants. This positive user experience translated into significantly greater adherence to the updated disinfection procedures, and could also have played a role in preventing a rise in CDI cases despite increased antibiotic use.

Antibiotic stewardship programs are critical in mitigating CDI outbreaks, as antibiotics disrupt the gut microbiome, facilitating *C. difficile* proliferation. Judicious antibiotic use is essential in preventing CDI, but its impact on CDI rates is complex. Some studies, including ours, have shown stable or decreased CDI incidence despite increased antibiotic consumption [51,52]. This highlights the multifaceted nature of CDI control, where factors beyond antibiotic use, such as comprehensive infection prevention measures, play a role.

Rational antibiotic prescribing ensures antibiotics are used only when clinically indicated, tailored to patient needs, and appropriately dosed and timed, minimizing the risk of CDI [53]. Additionally, infection control strategies, including enhanced cleaning, contact precautions, hand hygiene, and rapid diagnostics, are crucial. The absence of a CDI increase, despite higher antibiotic use, likely reflects the success of a comprehensive infection control program integrating these measures.

## 5. Limitations and Strength of the Study

Limitations of this study include the before–after design, without a concurrent control unit or randomization. Thus, one cannot rule out the possibility that other factors may partly explain the difference in the CDI rate. However, no new infection control initiatives were implemented during this period. This was a single-center retrospective study. Moreover, the effect of proton pump inhibitors on the incidence of *C. difficile* infections was not evaluated.

## 6. Conclusions

In conclusion, the absence of a CDI surge despite increased antibiotic consumption highlights the synergistic relationship between antibiotic stewardship and rigorous infection control practices. The combination of the improved disinfection protocol and comprehensive staff training proved remarkably effective in mitigating CDI risk. This emphasizes the importance of investing in multifaceted strategies which include both antibiotic stewardship and non-antibiotic interventions as key elements in reducing healthcare-associated infections. Future initiatives should focus on continuing to improve and expand these strategies to maintain low rates of CDI infection within healthcare settings. Adequate resources, such as PPE, cleaning materials, finance, and educated employees, are all required for effective infection control. In the absence of these resources, implementing and maintaining effective infection control may be difficult [54,55].

## Figures and Tables

**Table 1:** Monthly distribution of registered *C. difficile* infections in the Anesthesiology Department across three observation periods.

Characteristic	01.2019–07.2022, N = 43 ^1^	08.2022–02.2023, N = 7 ^1^	03.2023–10.2023, N = 8 ^1^	*p*-Value ^2^
Number of infections	months	months	months	0.79
0	27 (63%)	5 (71%)	5 (63%)	
1	12 (28%)	1 (14%)	3 (38%)	
2	4 (9.3%)	1 (14%)	0 (0%)	

^1^ n (%); ^2^ Fisher’s exact test.

**Table 2:** Monthly distribution of registered *C. difficile* infections in the Gastroenterology Department across three observation periods.

Characteristic	01.2019–07.2022, N = 43 ^1^	08.2022–02.2023, N = 7 ^1^	03.2023–10.2023, N = 8 ^1^	*p*-Value ^2^
Number of infections	months	months	months	0.10
0	17 (40%)	3 (43%)	8 (100%)	
1	13 (30%)	4 (57%)	0 (0%)	
2	8 (19%)	0 (0%)	0 (0%)	
3	1 (2.3%)	0 (0%)	0 (0%)	
4	4 (9.3%)	0 (0%)	0 (0%)	

^1^ n (%); ^2^ Fisher’s exact test.

**Table 3:** Monthly distribution of registered *C. difficile* infections in the Internal Medicine Department across three observation periods.

Characteristic	01.2019–07.2022, N = 43 ^1^	08.2022–02.2023, N = 7 ^1^	03.2023–10.2023, N = 8 ^1^	*p*-Value ^2^
Number of infections	months	months	months	0.77
0	24 (56%)	3 (43%)	3 (38%)	
1	10 (23%)	2 (29%)	3 (38%)	
2	5 (12%)	2 (29%)	2 (25%)	
3	3 (7.0%)	0 (0%)	0 (0%)	
4	1 (2.3%)	0 (0%)	0 (0%)	

^1^ n (%); ^2^ Fisher’s exact test.

**Table 4:** Antibiotic consumption in the gastroenterology clinic during the study period expressed in DDS/1000PD.

Characteristics	Before Intervention N = 43	Intervention Period N = 7	After Intervention N = 8
Fluoroquinolones			
Mean (SD)	186.5 (57.0)	148.6 (42.5)	220.4 (46.7)
Median	177.1	143.4	233.4
IQR	[143.2, 210.5]	[130.7, 172.4]	[202.3, 254.0]
Min–Max	92.3–331.0	81.8–208.7	122.9–265.9
Cephalosporins			
Mean (SD)	78.0 (37.5)	71.2 (36.8)	118.5 (42.5)
Median	73.8	57.6	116.4
IQR	[54.7, 98.7]	[53.5, 81.5]	[89.9, 149.0]
Min–Max	0.0–176.4	27.8–143.0	60.6–181.5
Amoxicillin			
Mean (SD)	7.7 (9.3)	6.6 (5.4)	3.3 (7.8)
Median	4.4	7.1	0.0
IQR	[0.0, 13.1]	[2.1, 11.2]	[0.0, 0.9]
Min–Max	0.0–33.8	0.0–12.6	0.0–22.4
Clindamycin			
Mean (SD)	0.2 (0.4)	2.2 (5.8)	4.8 (10.7)
Median	0.0	0.0	0.0
IQR	[0.0, 0.0]	[0.0, 0.0]	[0.0, 2.1]
Min–Max	0.0–1.6	0.0–15.5	0.0–30.3
Carbapenems			
Mean (SD)	4.6 (2.5)	35.7 (28.5)	57.9 (46.3)
Median	4.2	26.7	51.0
IQR	[3.1, 5.4]	[17.3, 40.9]	[32.4, 64.4]
Min–Max	0.8–12.9	13.5–93.6	11.9–160.7

IQR—Interquartile Range.

**Table 5:** Antibiotic consumption in the Internal Medicine Department during the study period expressed in DDS/1000PD.

Characteristics	Before Intervention N = 43	Intervention Period N = 7	After Intervention N = 8
Fluoroquinolones			
Mean (SD)	171.4 (166.9)	136.4 (56.2)	123.2 (19.3)
Median	140.4	120.8	124.0
IQR	[117.1, 176.5]	[95.7, 178.4]	[116.3, 132.9]
Min–Max	24.0–1196.1	69.2–216.9	86.0–148.9
Cephalosporins			
Mean (SD)	247.3 (92.0)	213.5 (55.6)	203.3 (51.5)
Median	240.3	209.8	191.6
IQR	[192.6, 270.9]	[181.1, 229.2]	[168.4, 248.7]
Min–Max	72.6–574.2	147.9–316.0	138.6–274.9
Amoxicillin			
Mean (SD)	8.4 (12.8)	9.0 (14.7)	4.9 (8.1)
Median	5.8	6.0	1.0
IQR	[0.0, 11.9]	[1.2, 6.6]	[0.0, 5.4]
Min–Max	0.0–62.3	0.0–41.6	0.0–22.7
Clindamycin			
Mean (SD)	1.4 (1.9)	16.9 (15.7)	20.5 (17.2)
Median	0.7	15.5	21.5
IQR	[0.0, 2.2]	[4.8, 25.2]	[6.9, 33.2]
Min–Max	0.0–8.8	0.0–43.0	0.0–44.8
Carbapenems			
Mean (SD)	6.0 (2.3)	59.6 (23.5)	75.6 (24.3)
Median	5.6	67.2	80.1
IQR	[4.4, 6.8]	[41.1, 75.6]	[54.6, 83.3]
Min–Max	2.3–12.6	27.0–89.2	45.1–121.1

IQR—Interquartile Range.

**Table 6:** Antibiotic consumption in the Department of Anesthesiology and Intensive Care during the study period expressed in DDS/1000PD.

Characteristics	Before Intervention N = 43	Intervention Period N = 7	After Intervention N = 8
Fluoroquinolones			
Mean (SD)	63.0 (41.4)	81.1 (61.9)	55.9 (23.6)
Median	55.7	51.9	58.6
IQR	[31.2, 96.4]	[42.4, 92.9]	[40.3, 73.2]
Min–Max	0.0–172.0	36.5–208.8	15.9–86.7
Cephalosporins			
Mean (SD)	91.4 (48.6)	130.3 (44.0)	145.6 (40.0)
Median	73.1	108.7	147.5
IQR	[60.8, 132.9]	[102.5, 149.2]	[119.7, 183.2]
Min–Max	0.0–188.4	86.8–213.1	87.0–189.3
Amoxicillin			
Mean (SD)	31.6 (30.0)	18.2 (26.2)	6.9 (13.4)
Median	27.4	0.0	0.0
IQR	[5.5, 44.3]	[0.0, 29.4]	[0.0, 5.3]
Min–Max	0.0–126.4	0.0–68.3	0.0–36.1
Clindamycin			
Mean (SD)	6.1 (4.9)	77.5 (51.2)	100.8 (40.9)
Median	5.0	76.8	102.4
IQR	[2.7, 8.2]	[34.4, 109.0]	[70.0, 113.1]
Min–Max	0.0–20.4	19.9–159.4	49.9–176.6
Carbapenems			
Mean (SD)	13.4 (6.8)	159.4 (85.3)	214.6 (57.9)
Median	12.9	171.8	208.0
IQR	[8.1, 16.1]	[116.8, 188.6]	[165.0, 257.8]
Min–Max	2.5–35.0	31.9–301.4	144.5–301.7

IQR—Interquartile Range.

**Table 7:** Overall change in antibiotic consumption in the study period.

	Beta	95% CI ^1^	*p*-Value
**Fluoroquinolones**			
Before intervention	—	—	
Intervention period	−18.3	−61.9, 25.3	0.4
After intervention	−7.2	−48.4, 34.0	0.7
**Clindamycin**			
Before intervention	—	—	
Intervention period	29.7	19.9, 39.5	<0.001
After intervention	39.5	30.2, 48.7	<0.001
**Cephalosporins**			
Before intervention	—	—	
Intervention period	−0.5	−29.2, 28.1	>0.9
After intervention	16.9	−10.2, 44.0	0.2
**Amoxicillin**			
Before intervention	—	—	
Intervention period	−4.6	−13.3, 4.0	0.3
After intervention	−10.9	−19.1, −2.7	0.009
**Carbapenems**			
Before intervention	—	—	
Intervention period	76.9	60.4, 93.4	<0.001
After intervention	108.0	92.4, 123.7	<0.001

^1^ CI = Confidence Interval.

## Data Availability

The original contributions presented in this study are included in the article/Supplementary Materials. Further inquiries can be directed to the corresponding author.

## Supplementary Materials

The following supporting information can be downloaded at: https://www.mdpi.com/article/10.3390/jcm14144904/s1, High-touch Room Surfaces.
