# Supplementary material for: Impact of an Enhanced Disinfection Protocol on the Incidence of Clostridioides difficile Infections and Antibiotic Consumption in a Hospital Setting: A Retrospective Intervention Study
Source: J Clin Med. 2025 Jul 10;14(14):4904. doi: 10.3390/jcm14144904 (PMC12296126; doi:10.3390/jcm14144904)
Supplement: Supplementary file 1 [file jcm-14-04904-s001.zip › jcm-3618668-supplementary.pdf]

High-touch Room Surfaces

Bed rails / controls

Tray table

IV pole (grab area)

Call box / button

Telephone

Bedside table handle

Chair

Room sink

Room light switch

Room inner doorknob

Bathroom inner doorknob / plate

Bathroom light switch

Bathroom handrails by toilet

Bathroom sink

Toilet seat

Toilet flush handle

IV pump control

Multi-module monitor controls

Multi-module monitor touch screen
